# The effects of aqueous extract of *Aloe vera* leaves on the gastric acid secretion and brain and intestinal water content following acetic acid- induced gastric ulcer in male rats

**Published:** 2014

**Authors:** Zakieh Keshavarzi, Taha Mohammad Rezapour, Mehran Vatanchian, Mohammad Zare Hesari, Hadi Nabizade Haghighi, Mostafa Izanlu, Maryam Sabaghian, Kaveh Shahveisi

**Affiliations:** 1 *Department of Physiology, School of Medicine, Bojnurd University of Medical Sciences, Bojnurd, I. R. Iran*; 2 *Student Research Committee, School of Medicine, North Khorasan University of Medical Sciences, Bojnurd, I. R. Iran*; 3 *Department of Physiology, North Khorasan University of Medical Sciences, Bojnurd, I. R. Iran*; 4 *Department of Physiology, School of Medicine, Mashhad University of Medical Sciences, Mashhad, I. R. Iran*

**Keywords:** *Aloe vera*, *Brain water content*, *Gastric acid secretion*, *Peptic ulcer*

## Abstract

**Objective:** Gut–brain axis (GBA) is very important in creation and modulation of gastrointestinal problems.* Aloe vera* gel has gastroprotective properties. The purpose of this study was to evaluate the effect of aqueous extract of *Aloe vera *leaves on the gastric acid secretion and brain and intestinal water content following acetic acid gastric ulcer induction.

**Materials and Methods: **Gastric ulcer was induced by injection of 20% acetic acid into the subserosal layer in male rats. Rats were randomly assigned into three groups: intact group, gastric ulcer group and *Aloe vera* group (treatment with *Aloe vera* following gastric ulcer induction). The acid levels and brain and intestinal water content of each sample were measured eight days after the gastric ulcer induction.

**Results: **Gastric acid levels were significantly decreased in *Aloe vera* group when compared with gastric ulcer group (p<0.05). However, there were no differences in acid output between gastric ulcer and *Aloe vera* groups with intact group. After *Aloe vera* administration, the amount of brain water content had no difference with intact and gastric ulcer groups (p<0.05). The duodenal water content in *Aloe vera* group was significantly reduced compared with intact group (p<0.05) but gastric ulcer group had no significant difference with intact and *Aloe vera* group.

**Conclusions**: The administration of *Aloe vera* has an inhibitory effect on the gastric acid output.

## Introduction

Gut–brain axis (GBA) is very important in creation and modulation of gastrointestinal problems. It was described that GBA is a bidirectional neurohumoral communication system that integrates brain and gastrointestinal (GI) functions. The brain is the most influential organ within the axis. 

Peptic ulcer disease as gastrointestinal problem is created by imbalance pepsin and gastric acid secretion (Ramakrishnan, 2007 ▶; Labenz, 1994 ▶; Bureau, 2003 ▶; Joshi, 2004 ▶) and it is a common clinical problem that occurs mostly in the stomach and proximal duodenum (Cheng, 2000 ▶). Additionally, the vagus nerve has an important role in signaling from the GI tract to the brain and can be stimulated by endotoxins or inflammatory cytokines such as interleukin-1β and tumor necrosis factor α that are released in the peptic ulcer disease (Borovikova, 2000 ▶). Thus, it is better to consider the influence of the gut on the brain and vice versa.

Natural substances with therapeutic properties have been used in ancient times. Nowadays, a number of drugs prescribed originate from plants and some natural precursors (Rates, 2001 ▶). One of the materials used in a variety of medical conditions such as wounds healing and reduce tissue damages is the *Aloe Vera* plant (Vogler, 1999 ▶; Visuthikosol, 1995 ▶).

*Aloe vera* is a shrubby or arborescent, perennial, xerophytic, succulent, and pea-green color plant. It grows mainly in the dry regions of Africa, Asia, Europe, and America. It contains vitamins A (beta-carotene), C, and E, which are antioxidants. It provides calcium, copper, magnesium, potassium, and zinc which are essential for the proper functioning of various enzyme systems in different metabolic pathways and a few of them are antioxidants (Surjushe, 2008 ▶). The *aloe vera* is used in many situations such as wound healing. It is demonstrated that the extract of *Aloe vera* can effectively heal burns, skin damage, edema, and pain. It has been shown that the gel of this plant may protect humans and rodents from gastric ulcers. The extract also possesses anti-inflammatory properties, anti-diabetes, cellular protection, restoration, and mucus-stimulating activities (Davis, 1989 ▶; Parish, 1991 ▶; Klein, 1988 ▶; Lushbaugh, 1953 ▶; Hamman, 2008 ▶).

Therefore, with regard to the importance of gut–brain axis as bidirectional neurohumoral communication system in pathogenesis of peptic ulcer disease and brain disorders and also an increase in people's tendency to use herbs, the purpose of this study was to investigate the effects of aqueous extract of *aloe vera* leaves on the gastric acid secretion and brain and duodenal water content following acetic acid-induced gastric ulcer in male rats.

## Materials and Methods

**Animals**

Twenty-one adult male Wistar rats (200 to 250 g) were purchased from Animal Center of Mashhad University of Medical Sciences, Iran. The rats were housed in temperature and humidity controlled animal quarters with a 12-hour light/dark cycle with free access to food and water. All procedures were approved by the Institutional Animal Care Committee of North Khorasan University of Medical Sciences and were in accordance with the guidelines of the National Institutes of Health on the care and use of animal’s surgery.

**Experimental groups**

The rats were randomly divided into three groups (n= 7 in each group):

1- Intact group: animals that were not given any drugs (they received distilled water freely).

2- Gastric ulcer group: animals without treatment.

3- *Aloe vera* group: gastric ulcer animals that were treated with oral administration of* Aloevera* (200 mg/kg/dose, twice daily for 8 days) (Eamlamnam, 2006 ▶).

**Preparation of aqueous extract of Aloe vera**

Whole fresh leaves of *Aloe vera *L. (A. barbadensis Miller) separated from locally available plant, identified by botanist of Ferdowsi university of Mashhad, Iran. Whole leaves of *Aloe vera* were cut into thin pieces, put onto a glass plate and then ground into a fine powder for further use. The dried powder (120 g) was mixed with distilled water in a balloon and was shaken for two days. The preparation was then filtered off using a Gauze mesh and the solvent was dried by evaporation under reduced pressure at 40 °C. The final product yielded an 8% w/w dried extract. 

**Induction of ulcer**

The animals were fasted but allowed only water 12 hours before experiment. On the day of experiment, the animals were anesthetized with ether. The animals were located on the surgical table (manufactured by Bojnurd University of Medical Sciences, Bojnurd, Iran). The abdominal wall at midline epigastric line was incised and the stomach was extended and fixed. After exposing the stomach, 20% acetic acid (0.02 ml) was injected into the subserosal layer of the glandular portion of the anterior wall with a microsyringe (0.05 ml). At the time of injection, a thumb was placed tightly on the inserted needle to avoid solution leakage. Accuracy of the injection was confirmed by observation of wheal-like swelling at the injection site. After closing the abdomen, the animals were routinely maintained (Susumu, 2005).

**Evaluation of gastric acid secretion**

After surgery, a period of 30 min was allowed for stabilization. The stomach was washed with saline before the start of the first collection to remove any residual secretion to obtain the basal secretion. Once the gastric acid secretion (gastric acid output) had been stable for 30 min, it was considered as basal acid secretion. Throughout the experiment, the gastric secretions were collected in consecutive 15-min samples. The acid content of each gastric washout sample was measured with a manual titrator to an endpoint pH of 7 with 0.01 N NaOH and expressed as μmol H^+^/15 min (Nabavizadeh, 2007 ▶).

**Determination of brain water content**

To measure the brain edema, the brain water content was determined as the animal was anesthetized and the brain was extracted 8 days after ulcer induction. The weight of wet tissue was measured first and then incubated in 60 °C in an incubator (Memmert, Germany) for 72 hours to evaporate the tissue water and become dry. The brain was weighed again after drying and water content was calculated by % using formula (O’Connor, 2005 ▶).

$\mathrm{\% Water content}=\left[\frac{\left(\mathrm{wet weight}-\mathrm{dry weight}\right)}{\left(\mathrm{wet weight}\right)}\right]\times 100$

**Determination of duodenal water content**

Representive sections of small intestine (duodenal part) were obtained, and their contents were removed. Wet weights of the intestinal walls were determined, then the samples were dried in a vacuum oven for 48 h at 60°C and weighted immediately to determine the dry weights. Water content of duodenum was calculated by % using formula (AkhtarS, 2009 ▶).

$\mathrm{\% Water content}=\left[\frac{\left(\mathrm{wet weight}-\mathrm{dry weight}\right)}{\left(\mathrm{wet weight}\right)}\right]\times 100$

**Statistical analysis**

SPSS 15 was used for statistical analysis. Each parameter was expressed as mean±SEM, and compared using one-way ANOVA analysis of variance, followed by a Tukey’s post-hoc test. Differences between the mean were considered statistically significant when p<0.05.

**Ethical considerations**

This study protocol was approved by the ethics review committee of Student Research Committee, North Khorasan University of Medical Sciences.

## Results

**Effect on gastric acid secretion**

**Figure 1:**
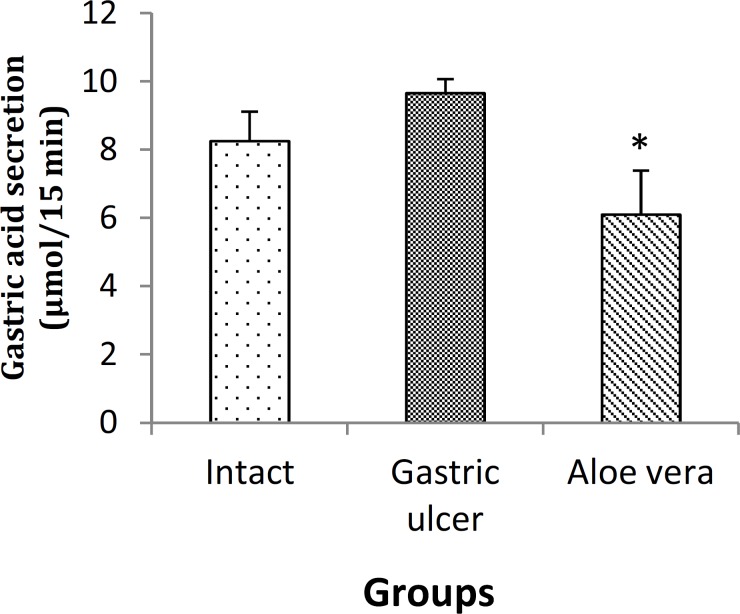
Acid output (µmol/15 min) in different groups after gastric ulcer induction. Data are presented as mean±SEM. *p<0.05, *Aloe vera *group *vs. *gastric ulcer group

Changes in gastric acid secretion levels (acid output) for different groups are shown in Figure 1. Gastric acid levels (acid output) were significantly decreased in *Aloe vera* (treatment) group (6.10±1.288 μmol/15 min) when compared with gastric ulcer group (9.66±0.411 μmol/15 min) (p<0.05). However, there were no differences in gastric acid levels between gastric ulcer and *Aloe vera* groups with intact group (8.25±0.869 μmol/15 min).

**Effect on brain water content**

**Figure 2:**
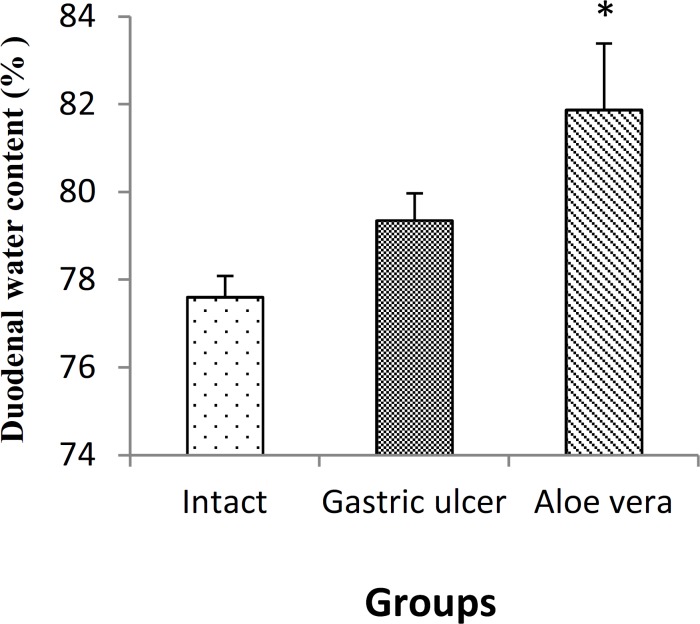
Alterations of brain water content, following gastric ulcer induction and treatment of gastric ulcer with *Aloe Vera* in male rats. Data are presented as mean±SEM. *p<0.05, gastric ulcer group *vs. *intact group

Figure 2 shows the brain water content in different groups of study. The amount of brain water content in gastric ulcer group (76.76±0.278%) was significantly reduced compared with intact group (78.50±0.643%) (p<0.05). After *Aloe vera* administration, the amount of brain water content was 77.06±0.582% but had no difference with intact and gastric ulcer groups (p<0.05).

**Effect on duodenal water content**

**Figure 3:**
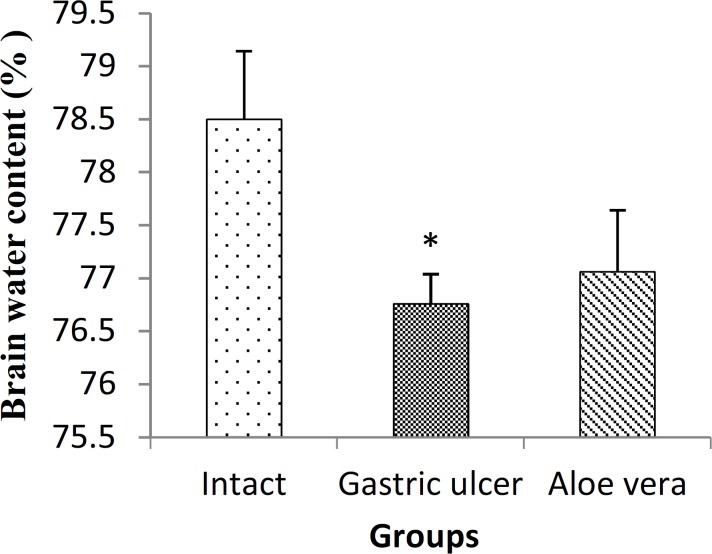
Alterations in duodenal water content following gastric ulcer induction and treatment of gastric ulcer with *Aloe Vera*. Data are presented as mean±SEM.*p<0.05, *Aloe vera *group *vs.* intact group

As shown in Figure 3, the duodenal water content in *Aloe vera* group (81.87±1.516%) was significantly reduced compared with intact group (78.50±0.643%) (p<0.05). However, gastric ulcer group (79.35±0.622%) had no significant difference with intact and *Aloe vera* groups.

## Discussion

Our results showed that *Aloe vera* extract inhibits gastric acid secretion. This may suggest that, at this dose, the plant may possess gastroprotective activity against gastric acid as an aggressive factor (Robert, 1979). The mechanism of cytoprotection is presently unknown. Several hypotheses have been suggested, such as increased mucus synthesis (Bolton et al., 1978; Kauffman et al., 1980), bicarbonate secretion (Garner and Heylings, 1979), increased mucosal blood flow (Konturekand Robert, 1982), and increased phospholipids mucosal coating (Lichtenberger et al., 1983 ▶) among others (D’Souza and Dhume, 1991).

The acid reducing properties of *Aloe vera *(Hennessee and Cook, 1994) may be shared with other anti-ulcer drugs (Muller et al., 1983; Sewing et al., 1983; Rabon and Michael, 1990). It is possible that the antiulcer effect of Aloe vera should be mediated through the decrease in aggressive factors and increase in protective factors which provide the advantages for patient with gastric ulcer. In addition, the *Aloe vera* has been demonstrated to have wound healing properties (Heggers et al., 1993; Davis et al., 1989b ▶; Chithra et al., 1998). The observation that *Aloe vera* extract inhibits acid secretion may be due to the presence of lectins in the plant (Blitz et al., 1963 ▶). Lectins are proteins/glycoproteins which are capable of recognizing and binding to carbohydrate moieties (Bardocz et al., 1995). It has been shown that lectins inhibit aminopyrine uptake by parietal cells (Healey et al., 1998). Thus, the ability of the extract to inhibit gastric acid output maybe as a result of direct action on the acid producing cells (Mehta et al., 1993). 

It was reported that *Aloe vera *could promote burn wound healing in rats (Somboonwong, 2000 ▶; Duansak, 2003 ▶; Davis, 1994 ▶). In addition, *Aloe vera* could induce angiogenesis in vivo (Moon, 1999 ▶), which plays an important role in wound healing. *Aloe vera* can result in reduced vasoconstriction and improve perfusion of gastric mucosal capillaries, thus promoting ulcer healing (Blitz, 1963 ▶; Grindlay, 1986 ▶).

There are some papers related to our manuscript, but in this study, the relationship of gastric acid and intestinal and brain water contents was measured. Evidence is increasing for the role of GBA in the pathogenesis of GI. The vagus nerve has an important role in signaling from the GI tract to the brain and can be stimulated by endotoxins or inflammatory cytokines such as interleukin-1β and tumor necrosis factor α (Borovikova, 2000 ▶). 

Imbalance between the sympathetic and parasympathetic outflow from the central nervous system has been reported in patients with GI and may be associated with behavioral change (Lindgren 1991 ▶, Lindgren 1993 ▶). For example, depression has been correlated with Crohn’s disease, stress, and ulcerative colitis in separate groups of patients (Maunder 2008 ▶). Specifically, Kipnis et al., showed that, in mice, a deficit in peripheral T cells can result in cognitive and behavioral impairment, although the origin of these cells, and whether they cross the blood-brain barrier or signal from the periphery to influence behavior, is not known (Kipnis, 2004 ▶).

Our results showed that induction of gastric ulcer decreased the brain water content. It seems that induction of gastric ulcer as a major stress can contract the blood vessels and decrease their water permeability. On the other hand, after *aloe vera* administration, it was shown that it doesn’t have any effect on the brain water content. It may be due to the fact that *Aloe vera* has failed to cross the blood-brain barrier and therefore has no effect on the brain water content. The induction of gastric ulcer induces the release of inflammatory factors in blood. These factors can reach brain and aggravate the condition. However, because the method of gastric ulcer induction was limited in subserosal layer, it seems this local injury was not severe enough to release biochemical factors that cause a severe inflammatory reaction in the brain and increase the brain water content. Conversely, the brain vasoconstriction was more dominant and thus reduced the brain water content. It seems that we need more researches about mechanism of brain permeability and role of inflammatory and non-inflammatory mediators. On the other hand, maintenance of the intestinal barrier function depends on the integrity of cellular plasma membrane, tight junctions as well as the elaboration of endothelial and epithelial secreting products (Nusrat et al, 2000 ▶). Gastric injury can make epithelial injury and increased intestinal mucosal permeability and some strong stimulators of tissue damage. Moreover, in this study, it was shown that* Aloe vera* increased the duodenal water content. It seems that *Aloe vera* have toxic effect on the duodenal water content in this dose. 

Owing to the increased intestinal permeability with gut barrier injury, the bacteria and lipopolysaccharides (LPS) can enter the systemic circulation through the portal vein and the mesenteric lymph, and result in sepsis and multiple organ dysfunction syndrome (MODS). The intestinal tract, therefore, is regarded as “initiator” of MODS and its potential pathogenicity has been given more attention (Davidson et al, 2004 ▶).

In general, authors confirmed that administration of *Aloe vera* has an inhibitory effect on the gastric acid output. The inhibitory effect of *Aloe vera* was mostly due to the component of the formula and the plant gastroprotective activity. 
